# Longitudinal observational study of resting-state brain functional connectivity changes in anorexia nervosa post-treatment

**DOI:** 10.1192/j.eurpsy.2025.1416

**Published:** 2025-08-26

**Authors:** S. Yennampelli, P. Holštajn Zemánková

**Affiliations:** 1 Department of Psychiatry, Masaryk University; 2 Department of Psychiatry, Masaryk University and University Hospital Brno, Brno, Czech Republic

## Abstract

**Introduction:**

Altered brain functional connectivity (FC) of patients with anorexia nervosa (AN) has been observed in the acute phase of the disorder, but it remains unclear to what extent these alterations recover during weight normalization.

**Objectives:**

In this study, we used resting-state functional imaging data collected during hospitalization and six months post-treatment to investigate longitudinal changes in FC patterns in patients with AN during weight normalization. Since a core feature of AN is the disconnect between body signals and the brain, we aimed to elucidate whether treatment led to changes in the FC of the anterior cingulate cortex (ACC), a part of the brain’s limbic system that plays a crucial role in integrating visceral, attentional, and emotional information.

**Methods:**

Seventeen women with restricting type AN underwent MRI scans and assessments to measure BMI and eating disorder symptomatology (Eating Disorder Inventory- EDI) upon hospital admission and six months after discharge. Functional imaging data were processed using seed-based connectivity analysis with ACC as a seed.

**Results:**

Longitudinal analyses showed a significant (p<0.05 FDR corrected) increased FC post-treatment between the ACC and the precuneus, specifically Brodmann area 7. Participants demonstrated significant BMI increases, although no significant changes were observed in clinical symptomatology assessed by EDI.

**Conclusions:**

The findings indicate greater FC between the ACC and the precuneus as patients with AN complete treatment and undergo weight normalization. The precuneus is integral to one’s sense of self, perceived information integration, and reaction to cues. Therefore, increased FC between the ACC and precuneus might improve proprioception, body awareness, and frequency of body image disturbances—all abnormalities central to AN.

**Disclosure of Interest:**

None Declared

